# Clinical behavior and outcomes of breast cancer in young women with germline *BRCA* pathogenic variants

**DOI:** 10.1038/s41523-021-00224-w

**Published:** 2021-02-12

**Authors:** Matteo Lambertini, Marcello Ceppi, Anne-Sophie Hamy, Olivier Caron, Philip D. Poorvu, Estela Carrasco, Albert Grinshpun, Kevin Punie, Christine Rousset-Jablonski, Alberta Ferrari, Shani Paluch-Shimon, Angela Toss, Claire Senechal, Fabio Puglisi, Katarzyna Pogoda, Jose Alejandro Pérez-Fidalgo, Laura De Marchis, Riccardo Ponzone, Luca Livraghi, Maria Del Pilar Estevez-Diz, Cynthia Villarreal-Garza, Maria Vittoria Dieci, Florian Clatot, Francois P. Duhoux, Rossella Graffeo, Luis Teixeira, Octavi Córdoba, Amir Sonnenblick, Arlindo R. Ferreira, Ann H. Partridge, Antonio Di Meglio, Claire Saule, Fedro A. Peccatori, Marco Bruzzone, Marie Daphne t’Kint de Roodenbeke, Lieveke Ameye, Judith Balmaña, Lucia Del Mastro, Hatem A. Azim

**Affiliations:** 1 Department of Internal Medicine and Medical Specialties (DIMI), School of Medicine, University of Genova, Genova, Italy; 2 Department of Medical Oncology, U.O.C, Clinica di Oncologia Medica, IRCCS Ospedale Policlinico San Martino, Genova, Italy; 3 Clinical Epidemiology Unit, IRCCS Ospedale Policlinico San Martino, Genova, Italy; 4 Department of Medical Oncology, Institut Curie, Paris, France; 5 Department of Medical Oncology, Institut Gustave Roussy, Université Paris-Saclay, Villejuif, France; 6 Department of Medical Oncology, Dana-Farber Cancer Institute, Boston, MA USA; 7 Hereditary Cancer Genetics Group, Vall d’Hebron Institute of Oncology (VHIO), Hospital Universitari Vall d’Hebron Barcelona Hospital Campus, Barcelona, Spain; 8 Sharett Institute of Oncology, Hadassah-Hebrew University Medical Center, Jerusalem, Israel; 9 Department of General Medical Oncology and Multidisciplinary Breast Centre, Leuven Cancer Institute, University Hospitals Leuven, Leuven, Belgium; 10 Department of Surgery, Centre Léon Bérard, Lyon, France; 11 Department of Surgical Sciences, General Surgery III - Breast Surgery, Fondazione IRCCS Policlinico San Matteo, University of Pavia, aBRCAdaBRA onlus, Pavia, Italy; 12 Breast Oncology Unit, Shaare Zedek Medical Centre and Department of Oncology, Sheba Medical Center, Tel Hashomer, Jerusalem, Israel; 13 Department of Oncology and Haematology, Azienda Ospedaliero-Universitaria Policlinico di Modena, Modena, Italy; 14 Cancer Genetics Unit, Bergonie Institute, Bordeaux, France; 15 Department of Medical Oncology, Centro di Riferimento Oncologico di Aviano (CRO) IRCCS, Aviano, Italy; 16 Department of Medicine, University of Udine, Udine, Italy; 17 Department of Breast Cancer and Reconstructive Surgery, Maria Sklodowska-Curie National Research Institute of Oncology, Warsaw, Poland; 18 Department of Medical Oncology, INCLIVA University Hospital of Valencia, CIBERONC, Valencia, Spain; 19 Division of Medical Oncology, Department of Radiological, Oncological and Pathological Sciences, “La Sapienza” University of Rome, Rome, Italy; 20 Gynecological Oncology, Candiolo Cancer Institute, FPO – IRCCS, Candiolo, Turin, Italy; 21 Medical Oncology Unit, ASST Papa Giovanni XXIII, Bergamo, Italy; 22 University of Siena, Siena, Italy; 23 Departament of Oncology, Instituto do Cancer do Estado de Sao Paulo – Faculdade de Medicina da Universidade de Sao Paulo, Pacaembu, Sao Paulo Brazil; 24 Department of Research and Breast Tumors, Mexican National Cancer Institute, Mexico City, Mexico; 25 Breast Cancer Center, Hospital Zambrano Hellion, Tecnologico de Monterrey, San Pedro Garza Garcia, NL Mexico; 26 Department of Surgery, Oncology and Gastroenterology, University of Padua, Padua, Italy; 27 Medical Oncology 2, Veneto Institute of Oncology IOV - IRCCS, Padua, Italy; 28 Department of Medical Oncology, Centre Henri Becquerel, Rouen, France; 29 Department of Medical Oncology, Breast Clinic, Cliniques Universitaires Saint-Luc, UCLouvain, Brussels, Belgium; 30 Breast Unit of Southern Switzerland (CSSI), Oncology Institute of Southern Switzerland, Bellinzona, Switzerland; 31 Breast Disease Unit, Saint-Louis Hospital, APHP, Université de Paris, INSERM U976, Paris, France; 32 Obstetrics and Gynecology Department, Hospital Universitari Son Espases, Palma, Spain; 33 Oncology Division, Tel Aviv Sourasky Medical Center and Sackler Faculty of Medicine, Tel Aviv, Israel; 34 Breast Unit, Champalimaud Clinical Center, Champalimaud Foundation, Lisbon, Portugal; 35 Predictive Biomarkers and New Therapeutic Strategies in Oncology, INSERM Unit 981, Gustave Roussy, Villejuif, France; 36 Department of Genetics, Institut Curie, Paris, France; 37 Gynecologic Oncology Department, European Institute of Oncology IRCCS, Milan, Italy; 38 Department of Medicine, Institut Jules Bordet and Université Libre de Bruxelles (U.L.B.), Brussels, Belgium; 39 Data Centre, Institut Jules Bordet and Université Libre de Bruxelles (U.L.B.), Brussels, Belgium; 40 Breast Unit, IRCCS Ospedale Policlinico San Martino, Genova, Italy

**Keywords:** Breast cancer, Cancer genetics

## Abstract

Young breast cancer (BC) patients carrying a germline *BRCA* pathogenic variant (*mBRCA*) have similar outcomes as non-carriers. However, the impact of the type of gene (*BRCA1* vs. *BRCA2*) and hormone receptor status (positive [HR+] vs. negative [HR−]) on clinical behavior and outcomes of *mBRCA* BC remains largely unknown. This is an international, multicenter, hospital-based, retrospective cohort study that included *mBRCA* patients diagnosed, between January 2000 and December 2012, with stage I–III invasive early BC at age ≤40 years. From 30 centers worldwide, 1236 young *mBRCA* BC patients were included. Among 808 and 428 patients with *mBRCA1* or *mBRCA2*, 191 (23.6%) and 356 (83.2%) had HR+tumors, respectively (*P* < 0.001). Median follow-up was 7.9 years. Second primary BC (*P* = 0.009) and non-BC malignancies (*P* = 0.02) were more frequent among *mBRCA1* patients while distant recurrences were less frequent (*P* = 0.02). Irrespective of hormone receptor status, *mBRCA1* patients had worse disease-free survival (DFS; adjusted HR = 0.76, 95% CI = 0.60–0.96), with no difference in distant recurrence-free interval (DRFI) and overall survival (OS). Patients with HR+ disease had more frequent distant recurrences (*P* < 0.001) and less frequent second primary malignancies (BC: *P* = 0.005; non-BC: *P* = 0.18). No differences in DFS and OS were observed according to hormone receptor status, with a tendency for worse DRFI (adjusted HR = 1.39, 95% CI = 0.94–2.05) in patients with HR+ BC. Type of *mBRCA* gene and hormone receptor status strongly impact BC clinical behavior and outcomes in *mBRCA* young patients. These results provide important information for patients’ counseling on treatment, prevention, and surveillance strategies.

## Introduction

In women aged ≤40 years, breast cancer is the most common malignancy and the first cause of cancer-related mortality^1^. Despite the higher risk of developing triple-negative and HER2-positive breast cancer, the majority of breast malignancies arising in young patients are hormone receptor-positive tumors^2^. In young patients, the prognosis for hormone receptor-positive breast cancers is worse when compared to their older counterparts^3^.

Other age-related issues should be considered in the management of breast cancer in young women^4^. Among them, genetic counseling and testing is key. Approximately 12% of cases arising in women aged ≤40 years are related to germline pathogenic variants in *BRCA1* or *BRCA2*^5,6^. Indeed, young age at diagnosis is a criterion to refer patients to genetic counseling irrespective of family history or other tumor biological features^4^. Identification of a germline pathogenic variant in the *BRCA* genes plays a crucial role in cancer prevention and treatment^7,8^. *BRCA-*related breast cancers have distinct biological features, including a tendency for hormone receptor-negativity in *BRCA1* carriers and hormone receptor positivity in *BRCA2* carriers^9–12^.

Several studies have investigated the prognostic role of carrying germline *BRCA* pathogenic variants^13^. Compared to women with sporadic breast cancer, current evidence does not support different outcomes in those with *BRCA* pathogenic variants^13^. Similarly, no difference in survival outcomes between young breast cancer patients with or without germline *BRCA* pathogenic variants have been shown, except a trend for a survival advantage in *BRCA-*mutated patients with triple-negative breast cancer compared with non-carriers^6^. Nevertheless, besides the known differences in clinicopathological features in breast cancer cases associated with germline *BRCA1* or *BRCA2* pathogenic variants^9–12^, there is a lack of evidence so far on whether the type of mutated gene may also lead to potential differences in breast cancer clinical behavior and outcomes. In addition, the prognostic value of hormone receptor status in *BRCA-*related breast cancers remains largely unknown. We addressed these important issues in a large series of young breast cancer patients harboring germline *BRCA* pathogenic variants.

## Results

### Study cohort

Out of 1424 patients registered in the study from 30 centers worldwide, 1236 young *BRCA*-mutated breast cancer patients were eligible for inclusion in the present analysis (Supplementary Fig. 1).

Among 808 and 428 patients with germline *BRCA1* and *BRCA2* pathogenic variants, 191 (23.6%) and 356 (83.2%) had hormone receptor-positive tumors, respectively (*P* < 0.001).

### Comparison between patients with germline *BRCA1* and *BRCA2* pathogenic variants

Compared to patients in the *BRCA2* cohort, those in the *BRCA1* cohort were younger, more likely to have Israeli origin, had more grade 3 tumors, less lobular histology, nodal involvement, and HER2-positive disease (Table 1). Women in the *BRCA1* cohort received chemotherapy more frequently and, among those with hormone receptor-positive disease, fewer patients received adjuvant endocrine therapy (Table 1). Although patients in the *BRCA1* cohort underwent breast-conserving surgery more often, no difference was observed in rates of risk-reducing mastectomy or salpingo-oophorectomy between the *BRCA1* and *BRCA2* cohorts, respectively (Supplementary Table 1).

**Table 1:** Patient, tumor and treatment characteristics.

	*BRCA1* cohort N (%) *n* = 808	*BRCA2* cohort N (%) *n* = 428	*P* value^a^
Country			0.001
Europe	611 (75.6)	611 (75.6)	
North America	52 (6.4)	27 (6.3)	
Latin America	34 (4.2)	13 (3.0)	
Israel	111 (13.7)	28 (6.5)	
Year at diagnosis			0.33
2000–2004	167 (20.7)	97 (22.7)	
2005–2008	304 (37.6)	143 (33.4)	
2009–2012	337 (41.7)	188 (43.9)	
Age at diagnosis, median (IQR) years	34 (30–37)	36 (33–38)	<0.001
Age at diagnosis			<0.001
≤30 years	206 (25.5)	64 (15.0)	
31–35 years	281 (34.8)	143 (33.4)	
36–40 years	321 (39.7)	221 (51.6)	
Histology			<0.001
Ductal carcinoma	645 (79.8)	346 (80.8)	
Lobular carcinoma	10 (1.2)	29 (6.8)	
Others	87 (10.8)	20 (4.7)	
Missing	66 (8.2)	33 (7.7)	
Tumor grade			<0.001
G1	10 (1.2)	13 (3.0)	
G2	110 (13.6)	140 (32.7)	
G3	638 (79.0)	251 (58.6)	
Missing	50 (6.2)	24 (5.6)	
Tumor size			0.95
T1 (≤2 cm)	331 (41.0)	176 (41.1)	
T2-T3-T4 (>2 cm)	459 (56.8)	247 (57.7)	
Missing	18 (2.2)	5 (1.2)	
Nodal status			<0.001
N0	484 (59.9)	183 (42.8)	
N1-N2-N3	311 (38.5)	240 (56.1)	
Missing	13 (1.6)	5 (1.2)	
Hormone receptor status			<0.001
ER and/or PR positive	191 (23.6)	356 (83.2)	
ER and PR negative	617 (76.4)	72 (16.8)	
HER2 status			<0.001
HER2 negative	760 (94.1)	380 (88.8)	
HER2 positive	28 (3.5)	40 (9.3)	
Missing	20 (2.5)	8 (1.9)	
Breast surgery			<0.001
Breast-conserving surgery	428 (53.0)	158 (36.9)	
Mastectomy	372 (46.0)	264 (61.7)	
Missing	8 (1.0)	6 (1.4)	
Use of chemotherapy			<0.001
No	24 (3.0)	33 (7.7)	
Yes	782 (96.8)	395 (92.3)	
Missing	2 (0.2)	0 (0.0)	
Type of chemotherapy^b^			0.63
Anthracycline- and taxane-based	526 (67.3)	263 (66.6)	
Anthracycline-based	196 (25.1)	110 (27.8)	
Taxane-based	20 (2.6)	11 (2.8)	
Others	19 (2.4)	6 (1.5)	
Missing	21 (2.7)	5 (1.3)	
Use of endocrine therapy^c^			<0.001
No	28 (14.7)	15 (4.2)	
Yes	160 (83.8)	339 (95.2)	
Missing	3 (1.6)	2 (0.6)	
Type of endocrine therapy^d^			0.38
Tamoxifen alone	71 (44.4)	145 (42.8)	
Tamoxifen + LHRHa	47 (29.4)	116 (34.2)	
LHRHa alone	5 (3.1)	3 (0.9)	
AI ± LHRHa	7 (4.4)	14 (4.1)	
Tamoxifen and AI (±LHRHa)	29 (18.1)	58 (17.1)	
Missing	1 (0.6)	3 (0.9)	
Duration of endocrine therapy, median (IQR) months	60 (28.5 to 60)	60 (50 to 60)	0.02

*IQR* interquartile range, *G* tumor grade, *T* tumor size, *N* nodal status, *ER* estrogen receptor, *PR* progesterone receptor, *LHRHa* luteinizing hormone-releasing hormone agonist, *AI* aromatase inhibitors.

^a^Calculated after exclusion of missing values.

^b^Calculated among patients who received chemotherapy.

^c^Calculated among patients with hormone receptor-positive breast cancer.

^d^Calculated among patients with hormone receptor-positive breast cancer who received endocrine therapy.

Supplementary Tables 2 and 3 report the comparison in clinicopathological characteristics and risk-reducing surgery between the *BRCA1* and *BRCA2* cohorts according to hormone receptor status.

Median follow-up was 7.9 years (interquartile range 5.6–10.6 years), with no difference between the *BRCA1* and *BRCA2* cohorts (*P* = 0.95). In terms of the first DFS event, second primary malignancies (breast cancer: 17.0% vs. 12.2%, *P* = 0.009; non-breast cancer: 4.3% vs. 1.9%, *P* = 0.02) were more frequent in the *BRCA1* cohort while distant recurrences were less frequent (10.4% vs. 15.4%, *P* = 0.02) as compared to the *BRCA2* cohort (Table 2). When the pattern of the first DFS event was assessed between the *BRCA1* and *BRCA2* cohorts according to hormone receptor status, the only difference was a higher rate of second non-breast cancer primary malignancies (5.2% vs. 1.4%, *P* = 0.005) in the *BRCA1* cohort with hormone receptor-positive disease, being mostly ovarian cancer (Supplementary Table 4).

**Table 2:** Pattern of invasive disease-free survival events according to a type of BRCA-mutated gene.

	*BRCA1* cohort N (%) *n* = 808	*BRCA2* cohort N (%) *n* = 428	*P* value^a^
Follow-up, median (IQR)	7.9 (5.4 to 10.6)	7.9 (6.0 to 10.6)	0.95
No events	492 (60.9)	262 (61.2)	0.76
Loco-regional recurrence	54 (6.7)	36 (8.4)	0.35
Distant recurrence ± loco-regional recurrence	84 (10.4)	66 (15.4)	0.02
Second primary malignancy	35 (4.3)	8 (1.9)	0.02
Ovarian cancer	18 (2.2)	1 (0.2)	
Other	7 (0.9)	4 (0.9)	
Missing	10 (1.2)	3 (0.7)	
Second primary breast cancer	137 (17.0)	52 (12.2)	0.009
Death without any disease-free survival event	6 (0.7)	4 (0.9)	0.79

*IQR* interquartile range.

^a^*P*-values for time-dependent events estimated by means of the Log-rank test.

**Fig. 1:**
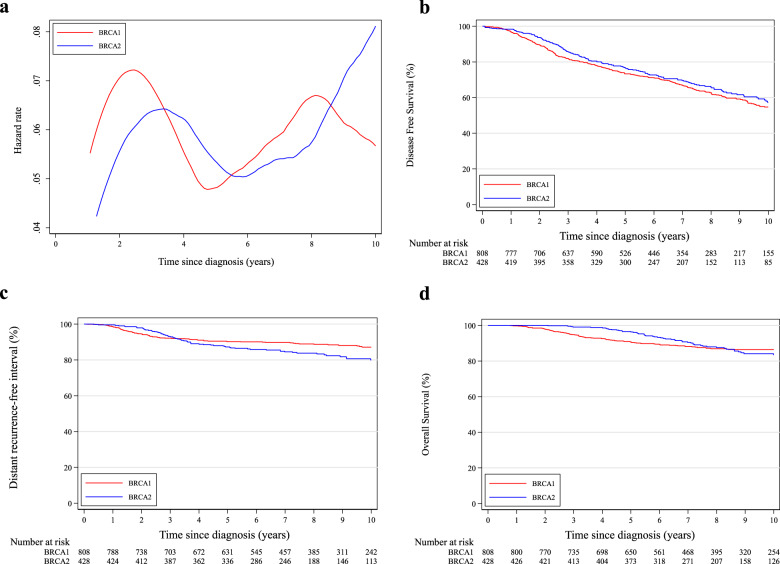
Comparison between patients with germline *BRCA1* and *BRCA2* pathogenic variants. **a** Epanechnikov Kernel-Smoothed annual hazards of recurrence overall; **b** Disease-free survival; **c** Distant recurrence-free interval; **d** Overall survival.

**Fig. 2:**
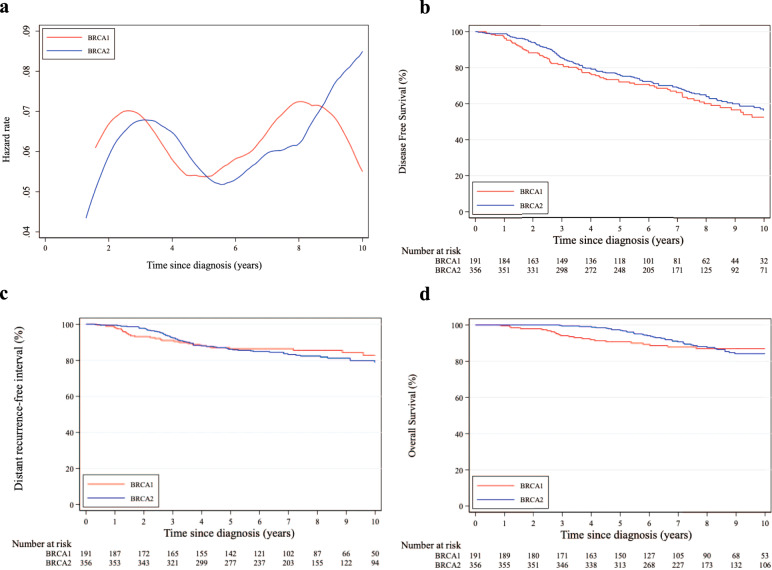
Comparison between patients with germline *BRCA1* and *BRCA2* pathogenic variants and hormone receptor-positive disease. **a** Epanechnikov Kernel-Smoothed annual hazards of recurrence overall; **b** Disease-free survival; **c** Distant recurrence-free interval; **d** Overall survival.

**Fig. 3:**
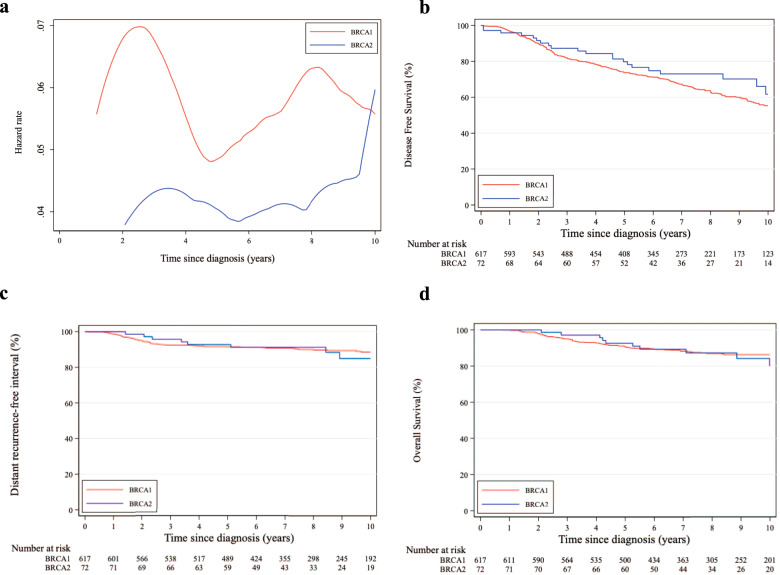
Comparison between patients with germline *BRCA1* and *BRCA2* pathogenic variants and hormone receptor-negative disease. **a** Epanechnikov Kernel-Smoothed annual hazards of recurrence overall; **b** Disease-free survival; **c** Distant recurrence-free interval; **d** Overall survival.

Considering DFS events, a similar pattern of annual HRs was observed between patients in the *BRCA1* and *BRCA2* cohorts irrespective of hormone receptor status, with a higher risk for those with *BRCA1* pathogenic variants (Figs. 1a, 2a, and 3a).

Eight-year DFS was 62.8% and 65.9% in the *BRCA1* and *BRCA2* cohorts, respectively (adjusted HR = 0.76, 95% CI 0.60–0.96; Fig. 1b). A tendency towards worse DFS in the *BRCA1* cohort was observed for both patients with hormone receptor-positive (adjusted HR = 0.77, 95% CI 0.58–1.03; Fig. 2b) and negative (adjusted HR = 0.73, 95% CI 0.48–1.13; Fig. 3b) breast cancer (*P*_interaction_ = 0.85; Supplementary Table 5).

Eight-year DRFI was 88.9% and 83.8% in the *BRCA1* and *BRCA2* cohorts, respectively (adjusted HR = 0.94, 95% CI 0.64–1.38; Fig. 1c). No significant difference in DRFI was observed for patients with either hormone receptor-positive (adjusted HR = 0.94, 95% CI 0.60–1.48; Fig. 2c) or negative (adjusted HR = 0.92, 95% CI 0.43–1.95; Fig. 3c) breast cancer (*P*_interaction_ = 0.95; Supplementary Table 6).

Eight-year OS was 86.9% and 87.5% in the *BRCA1* and *BRCA2* cohorts, respectively (adjusted HR = 0.69, 95% CI 0.46–1.04; Fig. 1d). No significant difference in OS was observed for patients with either hormone receptor-positive (adjusted HR = 0.64, 95% CI 0.39–1.05; Fig. 2d) or negative (adjusted HR = 0.80, 95% CI 0.40–1.56; Fig. 3d) breast cancer (*P*_interaction_ = 0.62; Supplementary Table 7).

### Comparison between patients with hormone receptor-positive and negative disease

Compared to patients with hormone receptor-negative disease, those with hormone receptor-positive breast cancer were older, had less grade 3 tumors, more often lobular histology, nodal involvement, and HER2-positive disease (Supplementary Table 8). Women with hormone receptor-positive breast cancer were less likely to receive chemotherapy and underwent mastectomy more often than those with hormone receptor-negative disease (Supplementary Table 8). No differences were observed in rates of risk-reducing mastectomy and salpingo-oophorectomy between patients with hormone receptor-positive and negative breast cancer, respectively (Supplementary Table 9).

**Fig. 4:**
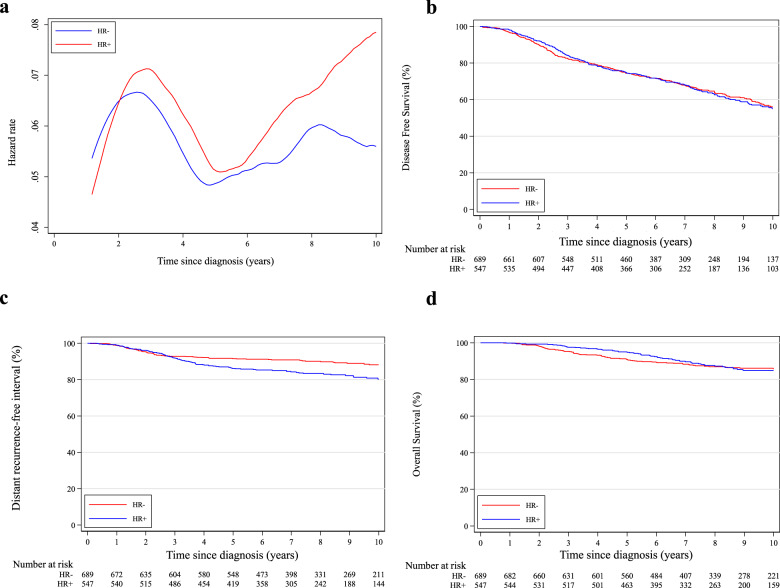
Comparison between patients with hormone receptor-positive and negative disease. **a** Epanechnikov Kernel-Smoothed annual hazards of recurrence overall; **b** Disease-free survival; **c** Distant recurrence-free interval; **d** Overall survival. HR+ hormone receptor-positive; HR− hormone receptor-negative.

In terms of first DFS event, patients with hormone receptor-positive breast cancer had a higher incidence of distant (±loco-regional) recurrences (16.1% vs. 9.0%, *P* < 0.001) and lower incidence of second primary malignancies (breast cancer: 12.1% vs. 17.9%, *P* = 0.005; non-breast cancer: 2.8% vs. 4.0%, *P* = 0.18) compared to women with hormone receptor-negative disease (Table 3). A similar pattern of annual HRs was observed between patients with hormone receptor-positive and negative disease until 5 years, beyond which a rapidly increasing trend for those with hormone receptor-positive breast cancer was observed (Fig. 4a).

**Table 3:** Pattern of invasive disease-free survival events according to hormone receptor status.

	Hormone receptor-positive N (%) *n* = 547	Hormone receptor-negative N (%) *n* = 689	*P* value^a^
Follow-up, median (IQR)	7.8 (5.8–10.6)	7.9 (5.5–10.6)	1.00
No events	329 (60.1)	425 (61.7)	0.91
Loco-regional recurrence	45 (8.2)	45 (6.5)	0.28
Distant recurrence +/− loco-regional recurrence	88 (16.1)	62 (9.0)	<0.001
Second primary malignancy	15 (2.8)	28 (4.0)	0.18
Ovarian cancer	7 (1.3)	12 (1.7)	
Other	6 (1.1)	5 (0.7)	
Missing	2 (0.4)	11 (1.6)	
Second primary breast cancer	66 (12.1)	123 (17.9)	0.005
Death without any disease-free survival event	4 (0.7)	6 (0.9)	0.75

*IQR* interquartile range.

^a^*P*-values for time-dependent events estimated by means of the Log-rank test.

As reported in Supplementary Table 10, 8-year DFS was 62.9% and 64.7% in patients with hormone receptor-positive and negative disease, respectively (adjusted HR = 1.06, 95% CI 0.84–1.33; Fig. 4b). Eight-year DRFI was 83.4% and 90.1% in patients with hormone receptor-positive and negative disease, respectively (adjusted HR = 1.39, 95% CI 0.94–2.05; Fig. 4c). Eight-year OS was 87.3% and 87.0% in patients with hormone receptor-positive and negative disease, respectively (adjusted HR = 0.81, 95% CI 0.55–1.20; Fig. 4d).

Considering that the cut-off used for defining hormone receptor positivity was not homogenous in all centers, the analyses comparing between patients with hormone receptor-positive and negative disease were then repeated by including only patients for whom the 1% cut-off for estrogen and/or progesterone receptor expression in their tumor was used to define hormone receptor status. Results were consistent with those reported in the main analyses (Supplementary Tables 11–14 and Supplementary Figs. 2–5).

## Discussion

This large unique dataset allowed an in-depth investigation of the impact of the type of mutated gene (*BRCA1* vs *BRCA2*) and hormone receptor status on clinical behavior and outcomes of *BRCA*-mutated breast cancer in young women. We observed that patients with germline *BRCA1* pathogenic variants had worse DFS than those with *BRCA2* pathogenic variants, mainly due to higher rates of second primary malignancies (predominantly breast and ovarian cancers) irrespective of hormone receptor status, with no difference in DRFI and OS. Hormone receptor positivity was not associated with favorable prognosis in *BRCA*-mutated breast cancer, whatever the type of mutated gene, but rather it displayed a tendency for worse DRFI and no difference in DFS and OS compared to hormone receptor-negative disease. These data are highly relevant for counseling young *BRCA*-mutated breast cancer patients.

In terms of clinicopathological features, as shown in prior studies^9–12^, the majority of breast cancer cases associated with germline *BRCA1* pathogenic variants were hormone receptor-negative tumors (76%) while those arising in *BRCA2-*mutated patients were hormone receptor-positive (83%). Overall, the majority of tumors were high-grade (60–80%) and HER2-negative (90–95%), both features being particularly relevant in the case of *BRCA1* carriers^9–12,14^. In terms of clinicopathological characteristics in the *BRCA1* and *BRCA2* cohorts according to hormone receptor status, the features that remained differently distributed were country of origin and tumor grade in patients with hormone receptor-positive disease (more likely Israeli origin and high-grade tumors in the *BRCA1* cohort) as well as age and HER2 status in those with hormone receptor-negative tumors (younger age and more likely HER2-negative status in the *BRCA1* cohort).

Harboring a germline pathogenic variant in *BRCA1* or *BRCA2* was not previously shown to have an apparent negative prognostic impact in breast cancer^13^, neither in young patients^6^. Compared with non-carriers, a tendency for better survival outcomes in *BRCA* carriers with triple-negative breast cancer was shown^6^. In addition, a reverse association of the prognostic value of hormone receptor status was observed between breast cancer patients with or without germline *BRCA* pathogenic variants^6,15^, particularly in the case of *BRCA2* pathogenic variants^16–18^. Nevertheless, within the cohort of young *BRCA-*mutated breast cancer patients, the prognostic implications of carrying a germline *BRCA1* or *BRCA2* pathogenic variant as well as potential differences according to hormone receptor status remained largely undefined. Two main findings with potential clinical implications were found in our study.

Firstly, irrespective of hormone receptor status, the type of *BRCA* gene does not appear to have prognostic value, with no observed difference in DRFI and OS between *BRCA1-* and *BRCA2-*mutated patients. This is reassuring evidence for counseling young *BRCA-*mutated breast cancer patients. Nevertheless, *BRCA1* carriers showed worse DFS, mostly due to higher rates of second primary malignancies (predominantly breast and ovarian cancers) as compared to *BRCA2*-mutated patients. This result reflects the different and age-dependent risk of cancer development associated with the two genes: the breast cancer peak of incidence occurs earlier and ovarian cancer risk is higher in *BRCA1* carriers as compared to those with *BRCA2* pathogenic variants^19,20^. Young age at cancer diagnosis further increases the risk of second primary breast and ovarian malignancies^19,21–23^. Therefore, these findings highlight the key role of *BRCA* genetic testing in this patient population and calls for awareness of the high risk of secondary malignancies and the option of considering risk-reducing surgeries when a pathogenic variant is identified, particularly in *BRCA1* carriers^24,25^. In addition, these results pinpoint the need to consider age-specific risk estimates when offering these procedures during patients’ counseling^23^.

Secondly, irrespective of the type of *BRCA*-mutated gene, hormone receptor positivity has no favorable prognostic value in this setting. While no difference in DFS and OS was observed, patients with hormone receptor-positive breast cancer showed a higher risk of distant relapses and a trend for worse DRFI as compared to those with hormone receptor-negative disease. There are several potential explanations for this lack of prognostic advantage in young *BRCA*-mutated patients with hormone receptor-positive breast cancer. Estrogen signaling was shown to promote tumor initiation and progression in *BRCA*-deficient cells^26,27^. Biological differences were reported between hormone receptor-positive breast cancers arising in patients with or without germline *BRCA* pathogenic variants^28,29^. When tested with OncotypeDx, a larger proportion of *BRCA*-mutated patients were found to have high recurrence scores, and low recurrence score was not necessarily associated with low risk of recurrence^30–32^. Taking into account that our study included only patients ≤40 years at diagnosis, these considerations may be further amplified due to the additional effect of young age on the biology of hormone-receptor-positive breast cancers^2,3^. Finally, it should be highlighted that *BRCA*-related tumors are known to have high chemosensitivity^11,17,33^, particularly in the case of hormone receptor-negative disease^34,35^. In our study, more than 90% of the patients received chemotherapy. However, ~8% of patients with hormone receptor-positive disease did not receive adjuvant endocrine therapy, with significantly higher numbers of non-recipients among *BRCA1* carriers. Although the reasons for lack of endocrine therapy administration were not collected, special attention should be paid in this regard when counseling *BRCA-*mutated patients with hormone receptor-positive disease. Our findings provide indirect evidence to potentially consider *BRCA-*mutated patients with hormone receptor-positive disease as a high-risk population^36^. Therefore, ovarian function suppression^37^ and extended adjuvant endocrine therapy^38^ may be considered relevant options in this setting. In light of the increased ovarian cancer risk, risk-reducing salpingo-oophorectomy might be considered as the primary strategy for ovarian function suppression in eligible patients who have completed their family planning.

The findings of this study should be considered in the context of its limitations. This is a retrospective cohort study conducted over a relatively long period of time. Assessment of *BRCA* and hormone receptor status as well as patient management were conducted according to diagnostic and treatment procedures available at that time in the respective country and center. Median follow-up was shorter than 10 years. Nevertheless, several unique features of this study should also be highlighted. This is a multicenter study, not restricted to a single continent, that included a large sample size despite focusing on a special and rare patient population (i.e., young breast cancer patients with germline *BRCA* pathogenic variants). The numbers of included patients and registered events made possible the acquisition of reliable results on these important unmet and clinically highly relevant issues.

In conclusion, type of *BRCA*-mutated gene and hormone receptor status strongly impact the clinical behavior and outcomes of breast cancer in young patients with germline *BRCA* pathogenic variants. Young patients with germline *BRCA1* pathogenic variants had worse DFS than those with germline *BRCA2* pathogenic variants mostly due to higher rates of second primary malignancies irrespective of hormone receptor status. On the other hand, unlike breast cancers arising in non-*BRCA* carriers, hormone receptor status had no prognostic value in young *BRCA*-mutated patients, and even a tendency of worse DRFI in women with hormone receptor-positive disease was observed. These results provide important information for counseling young *BRCA*-mutated breast cancer patients regarding treatment, prevention and surveillance strategies.

## Methods

### Study design and participants

Details of this study were previously reported^39^. Briefly, this was an international, multicenter, hospital-based, retrospective cohort study that included women diagnosed at age ≤40 years with invasive early breast cancer (stage I–III) between January 2000 and December 2012. All included patients had a known germline *BRCA1* or *BRCA2* pathogenic variant.

Healthy carriers as well as women with *BRCA* variants of uncertain significance, other malignancies (including ovarian cancer) without a prior diagnosis of invasive breast cancer, in situ or stage IV de novo breast cancer, or lack of information on follow-up were not eligible for inclusion. For the purpose of the present analysis, patients harboring pathogenic variants in both *BRCA1* and *BRCA2* as well as those with unknown hormone receptor status were also excluded.

Datasets from countries with more than one participating center were crosschecked to exclude potential duplicated cases.

### Procedures

Data on tumor and patient characteristics, treatment, *BRCA* pathogenic variants, and survival outcomes were collected for all eligible patients.

The type of mutated gene was the criteria used to distinguish between two cohorts of patients: women with *BRCA1* (*BRCA1* cohort) and those with *BRCA2* (*BRCA2* cohort) pathogenic variants.

*BRCA* pathogenic variants and hormone receptor status were assessed locally at each participating center. Hormone receptor positivity was defined by the expression of estrogen and/or progesterone receptors in ≥1% of invasive tumor cells (≥10% in French participating centers) assessed by immunostaining.

The Institut Jules Bordet (Brussels, Belgium) coordinated the study and acted as the central ethics committee. Ethics approval by the Institutional Review Boards of participating centers and patients’ written informed consent were obtained before inclusion whenever requested by local regulations.

The STrengthening the Reporting of OBservational studies in Epidemiology (STROBE) statement was followed for study reporting^40^.

### Outcomes

The current analysis aimed to investigate the impact of the type of mutated gene (*BRCA1* vs. *BRCA2*) and hormone receptor status on clinical behavior and outcomes of young breast cancer patients with germline *BRCA* pathogenic variants.

Clinicopathological characteristics, pattern, and risk over time of disease-free survival (DFS) events, as well as prognosis (in terms of DFS, distant recurrence-free interval [DRFI], and overall survival [OS]) were compared between the *BRCA1* and *BRCA2* cohorts. The same analyses comparing the *BRCA1* and *BRCA2* cohorts were then performed separately in patients with hormone receptor-positive and negative disease.

To specifically assess the effect of hormone receptor status, clinicopathological characteristics, pattern, and risk over time of DFS events, as well as prognosis (in terms of DFS, DRFI, and OS) were compared between patients with hormone receptor-positive and negative disease irrespective of the type of mutated gene.

### Statistical analysis

Descriptive analyses were used to assess clinicopathological characteristics as well as the pattern of DFS events.

To assess the risk of developing DFS events over time, the Epanechnikov Kernel-Smoothed annual hazards of recurrence were calculated.

DFS was defined as the time from diagnosis until the first appearance of one of the following invasive events: loco-regional recurrence, distant metastases, new contralateral or ipsilateral breast cancer, second primary malignancy, or death from any cause. DRFI was calculated as the time from diagnosis until the first occurrence of invasive breast cancer recurrence at a distant site. OS was defined as the time from diagnosis until death from any cause.

Observation times of patients that did not experience an event were censored on the date of their last contact. Kaplan–Meier plots were used to present results with a follow-up time of up to 10 years. Cox proportional hazard model was applied to estimate the hazard ratios (HRs) over the whole follow-up period, adjusting for the concomitant effect of selected confounders. Multivariate models for all survival analyses included nodal status, grade, HER2, type of breast surgery, chemotherapy use, age, year of diagnosis, and country.

Homogeneity tests on the HRs were performed in all survival analyses using the likelihood ratio test to assess whether there was evidence of an interaction between the type of gene and hormone receptor status.

All statistical analyses were two-sided; *P* values < 0.05 were considered as statistically significant. Statistical analyses were performed by MC and MB using Stata 13.1 (StataCorp. 2013. Stata Statistical Software: Release 13. College Station, TX: StataCorp LP).

### Reporting summary

Further information on research design is available in the Nature Research Reporting Summary linked to this article.

## Supplementary information

Online only data supplement

Reporting Summary Checklist

## Data Availability

The data generated and analysed during this study are described in the following data record: 10.6084/m9.figshare.13507422^41^. The data are stored in the following four Excel spreadsheets: ‘Patient survival data.xlsx’, ‘Patient baseline, tumor and treatment data.xlsx’, ‘Patient risk-reducing surgery data.xlsx’, ‘Patient eligibility criteria and survival data.xlsx’. These data are not publicly available for the following reason: data contain information that could compromise research participant privacy. However, the data can be made available upon reasonable request to the corresponding author. A list of which data file underlies which figure, table, and supplementary table in the related manuscript is provided in the file ‘Lambertini et al. underlying data lookup.csv’, included as part of the metadata record^41^. The dataset analysed during this study is described with more details in the following manuscript: 10.1200/JCO.19.02399^39^.

## References

[1] Fidler MM (2017). Cancer incidence and mortality among young adults aged 20-39 years worldwide in 2012: a population-based study. Lancet Oncol..

[2] Azim HA, Partridge AH (2014). Biology of breast cancer in young women. Breast Cancer Res..

[3] Partridge AH (2016). Subtype-dependent relationship between young age at diagnosis and breast cancer survival. J. Clin. Oncol..

[4] Paluch-Shimon, S. et al. ESO-ESMO 4th International Consensus Guidelines for Breast Cancer in Young Women (BCY4). *Ann. Oncol.* **31**, 674–696 (2020). 10.1016/j.annonc.2020.03.284 32199930

[5] Rosenberg SM (2016). BRCA1 and BRCA2 mutation testing in young women with breast cancer. JAMA Oncol..

[6] Copson ER (2018). Germline BRCA mutation and outcome in young-onset breast cancer (POSH): a prospective cohort study. Lancet Oncol..

[7] Paluch-Shimon S (2016). Prevention and screening in BRCA mutation carriers and other breast/ovarian hereditary cancer syndromes: ESMO Clinical Practice Guidelines for cancer prevention and screening. Ann. Oncol..

[8] Tung NM (2020). Management of Hereditary Breast Cancer: American Society of Clinical Oncology, American Society for Radiation Oncology, and Society of Surgical Oncology Guideline. J. Clin. Oncol..

[9] Eerola H (2005). Histopathological features of breast tumours in BRCA1, BRCA2 and mutation-negative breast cancer families. Breast Cancer Res..

[10] Atchley DP (2008). Clinical and pathologic characteristics of patients with BRCA-positive and BRCA-negative breast cancer. J. Clin. Oncol..

[11] Goodwin PJ (2012). Breast cancer prognosis in BRCA1 and BRCA2 mutation carriers: an International Prospective Breast Cancer Family Registry population-based cohort study. J. Clin. Oncol..

[12] Spurdle AB (2014). Refined histopathological predictors of BRCA1 and BRCA2 mutation status: a large-scale analysis of breast cancer characteristics from the BCAC, CIMBA, and ENIGMA consortia. Breast Cancer Res..

[13] van den Broek AJ, Schmidt MK, van’t Veer LJ, Tollenaar RAEM, van Leeuwen FE (2015). Worse breast cancer prognosis of BRCA1/BRCA2 mutation carriers: what’s the evidence? A systematic review with meta-analysis. PLoS ONE.

[14] Evans DG (2016). Low prevalence of HER2 positivity amongst BRCA1 and BRCA2 mutation carriers and in primary BRCA screens. Breast Cancer Res. Treat..

[15] Vocka, M. et al. Estrogen receptor status oppositely modifies breast cancer prognosis in BRCA1/BRCA2 mutation carriers versus non-carriers. *Cancers* **11**, 738 (2019). 10.3390/cancers11060738 PMC6627684 31141992

[16] Jonasson JG (2016). Oestrogen receptor status, treatment and breast cancer prognosis in Icelandic BRCA2 mutation carriers. Br. J. Cancer.

[17] Schmidt, M. K. et al. Breast cancer survival of BRCA1/BRCA2 mutation carriers in a hospital-based cohort of young women. *J. Natl. Cancer Inst*. **109** (2017). 10.1093/jnci/djw329 28376189

[18] Metcalfe K (2019). Oestrogen receptor status and survival in women with BRCA2-associated breast cancer. Br. J. Cancer.

[19] Kuchenbaecker KB (2017). Risks of breast, ovarian, and contralateral breast cancer for BRCA1 and BRCA2 mutation carriers. JAMA.

[20] Mavaddat N (2013). Cancer risks for BRCA1 and BRCA2 mutation carriers: results from prospective analysis of EMBRACE. J. Natl. Cancer Inst..

[21] Graeser MK (2009). Contralateral breast cancer risk in BRCA1 and BRCA2 mutation carriers. J. Clin. Oncol..

[22] Berkowitz Z, Rim SH, Peipins LA (2011). Characteristics and survival associated with ovarian cancer diagnosed as first cancer and ovarian cancer diagnosed subsequent to a previous cancer. Cancer Epidemiol..

[23] van den Broek AJ (2016). Impact of age at primary breast cancer on contralateral breast cancer risk in BRCA1/2 mutation carriers. J. Clin. Oncol..

[24] Hartmann LC, Lindor NM (2016). The role of risk-reducing surgery in hereditary breast and ovarian cancer. N. Engl. J. Med..

[25] Dullens, B. et al. Cancer Surveillance in Healthy Carriers of Germline Pathogenic Variants in BRCA1/2: A Review of Secondary Prevention Guidelines. *J. Oncol*. 9873954 (2020). 10.1155/2020/9873954 PMC7322604 32655641

[26] Baek HJ (2018). Inhibition of Estrogen Signaling Reduces the Incidence of BRCA1-associated Mammary Tumor Formation. Int. J. Biol. Sci..

[27] Wang C (2018). Estrogen promotes estrogen receptor negative BRCA1-deficient tumor initiation and progression. Breast Cancer Res..

[28] Tung N (2010). Estrogen receptor positive breast cancers in BRCA1 mutation carriers: clinical risk factors and pathologic features. Breast Cancer Res..

[29] Lips EH (2017). BRCA1-mutated estrogen receptor-positive breast cancer shows BRCAness, suggesting sensitivity to drugs targeting homologous recombination deficiency. Clin. Cancer Res..

[30] Lewin R (2016). Oncotype-DX recurrence score distribution in breast cancer patients with BRCA1/2 mutations. Breast Cancer Res. Treat..

[31] Shah PD (2016). Twenty-one-gene recurrence score assay in BRCA-associated versus sporadic breast cancers: differences based on germline mutation status. Cancer.

[32] Halpern N (2017). Oncotype Dx recurrence score among BRCA1/2 germline mutation carriers with hormone receptors positive breast cancer. Int. J. Cancer.

[33] Huzarski T (2013). Ten-year survival in patients with BRCA1-negative and BRCA1-positive breast cancer. J. Clin. Oncol..

[34] Jiang T (2016). Predictors of chemosensitivity in triple negative breast cancer: an integrated genomic analysis. PLoS Med..

[35] Tung N (2020). TBCRC 031: Randomized phase II study of neoadjuvant cisplatin versus doxorubicin-cyclophosphamide in germline BRCA carriers with HER2-negative breast cancer (the INFORM trial). J. Clin. Oncol..

[36] Lambertini M, Blondeaux E, Perrone F, Del, Mastro L (2020). Improving adjuvant endocrine treatment tailoring in premenopausal women with hormone receptor-positive breast cancer. J. Clin. Oncol..

[37] Burstein HJ (2016). Adjuvant Endocrine Therapy for Women With Hormone Receptor-Positive Breast Cancer: American Society of Clinical Oncology Clinical Practice Guideline Update on Ovarian Suppression. J. Clin. Oncol..

[38] Burstein HJ (2019). Adjuvant Endocrine Therapy for Women With Hormone Receptor-Positive Breast Cancer: ASCO Clinical Practice Guideline Focused Update. J. Clin. Oncol..

[39] Lambertini M (2020). Pregnancy after breast cancer in patients with germline BRCA mutations. J. Clin. Oncol..

[40] von Elm E (2007). The Strengthening the Reporting of Observational Studies in Epidemiology (STROBE) statement: guidelines for reporting observational studies. Lancet.

[41] Lambertini, M. et al. Metadata record for the manuscript: clinical behavior and outcomes of breast cancer in young women with germline BRCA pathogenic variants. figshare 10.6084/m9.figshare.13507422 (2020). 10.1038/s41523-021-00224-w PMC7880991 33579978

