# Supplementary material for: Clinical behavior and outcomes of breast cancer in young women with germline BRCA pathogenic variants
Source: NPJ Breast Cancer. 2021 Feb 12;7:16. doi: 10.1038/s41523-021-00224-w (PMC7880991; doi:10.1038/s41523-021-00224-w)
Supplement: Supplementary file 1 — Online only data supplement [file 41523_2021_224_MOESM1_ESM.pdf]

ONLINE DATA SUPPLEMENTS

Supplementary Figure 1. Study profile.

Supplementary Table 1. Use of prophylactic surgery comparing between patients with germline BRCA1 and *BRCA2* pathogenic variants.

Supplementary Table 2. Patient, tumor and treatment characteristics comparing between patients with germline *BRCA1* and *BRCA2* pathogenic variants according to hormone receptor status.

Supplementary Table 3. Use of prophylactic surgery comparing between patients with germline *BRCA1* and *BRCA2* pathogenic variants according to hormone receptor status.

Supplementary Table 4. Pattern of invasive disease-free survival events comparing between patients with germline *BRCA1* and *BRCA2* pathogenic variants according to hormone receptor status.

Supplementary Table 5. Disease-free survival comparing between patients with germline *BRCA1* and *BRCA2* pathogenic variants according to hormone receptor status.

Supplementary Table 6. Distant recurrence-free interval comparing between patients with germline *BRCA1* and *BRCA2* pathogenic variants according to hormone receptor status.

Supplementary Table 7. Overall survival comparing between patients with germline *BRCA1* and *BRCA2* pathogenic variants according to hormone receptor status.

Supplementary Table 8. Patient, tumor and treatment characteristics comparing between patients with hormone receptor-positive and negative breast cancer.

Supplementary Table 9. Use of prophylactic surgery comparing between patients with hormone receptor-positive and negative breast cancer.

Supplementary Table 10. Survival outcomes comparing between patients with hormone receptor-positive and negative breast cancer.

Supplementary Table 11. Patient, tumor and treatment characteristics comparing between patients with hormone receptor-positive and negative breast cancer (by including only patients for whom the 1% cut-off for estrogen and/or progesterone receptor expression in their tumor was used to define hormone receptor status).

Supplementary Table 12. Use of prophylactic surgery comparing between patients with hormone receptor-positive and negative breast cancer (by including only patients for whom the 1% cut-off for estrogen and/or progesterone receptor expression in their tumor was used to define hormone receptor status).

Supplementary Table 13. Pattern of invasive disease-free survival events according to hormone receptor status (by including only patients for whom the 1% cut-off for estrogen and/or progesterone receptor expression in their tumor was used to define hormone receptor status).

Supplementary Table 14. Survival outcomes comparing between patients with hormone receptor-positive and negative breast cancer (by including only patients for whom the 1% cut-off for estrogen and/or progesterone receptor expression in their tumor was used to define hormone receptor status).

Supplementary Figure 2. Comparison between patients with hormone receptor-positive and negative disease: Epanechnikov Kernel-Smoothed annual hazards of recurrence overall.

Supplementary Figure 3. Comparison between patients with hormone receptor-positive and negative disease: Disease-free survival.

Supplementary Figure 4. Comparison between patients with hormone receptor-positive and negative disease: Distant recurrence-free interval.

Supplementary Figure 5. Comparison between patients with hormone receptor-positive and negative disease: Overall survival.

This supplementary material has been provided by the authors to give readers additional information about their work.

Supplementary Figure 1. Study profile.

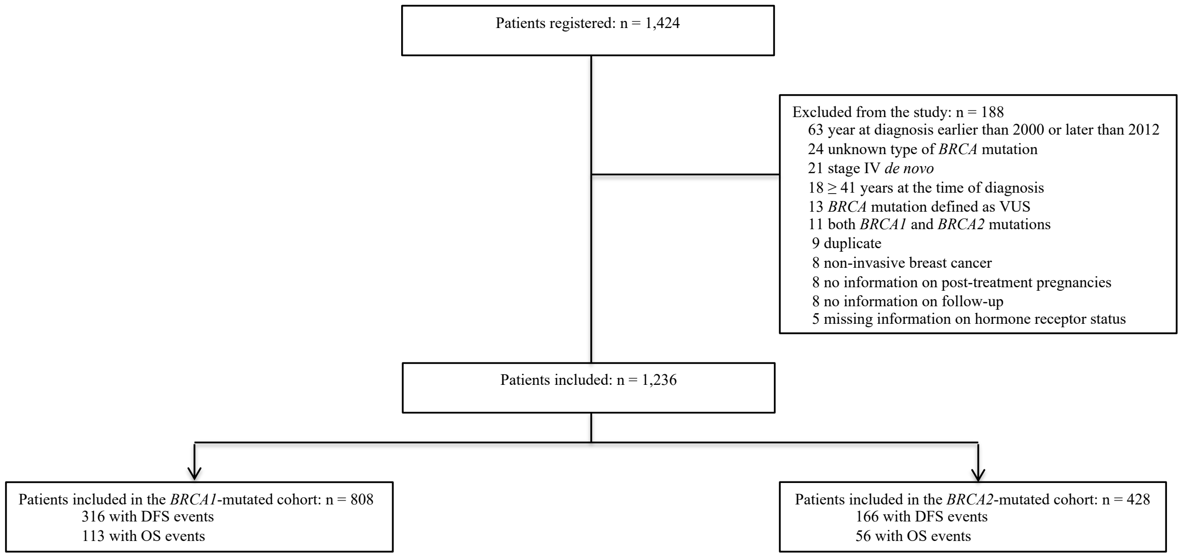

Abbreviations: VUS, variant of uncertain significance; DFS, disease-free survival; OS, overall survival.

Supplementary Table 1. Use of prophylactic surgery comparing between patients with germline *BRCA1* and *BRCA2* pathogenic variants.

	***BRCA1* cohort**<br>**N (%)**<br>**N=808**	***BRCA2* cohort**<br>**N (%)**<br>**N=428**	**P value[a]**
Prophylactic bilateral risk-reducing			0.371
mastectomy:	354 (43.8)	175 (40.9)	
No	355 (43.9)	197 (46.0)	
Yes	99 (12.2)	56 (13.1)	
Missing			
Prophylactic bilateral risk-reducing salpingo-oophorectomy:			1.000
No	328 (40.6)	176 (41.1)	
Yes	390 (48.3)	209 (48.8)	
Missing	90 (11.1)	43 (10.0)	
Date of prophylactic bilateral risk-reducing salpingo-oophorectomy:			
Not available, N (%)	441 (54.6)	232 (54.2)	0.905
Median, months (IQR)*	53.0 (19.6-92.5)	61.2 (26.1-96.3)	0.088

[a] Calculated after exclusion of missing values

*among patients with this information available

Supplementary Table 2. Patient, tumor and treatment characteristics comparing between patients with germline *BRCA1* and *BRCA2* pathogenic variants according to hormone receptor status.

	Hormone receptor-positive		P value[a]	Hormone receptor-negative		P value[a]
	BRCA1 **cohort N (%) N=191**	***BRCA2*** **cohort N (%) N=356**		***BRCA1*** **cohort N (%) N=617**	***BRCA2*** **cohort N (%) N=72**	
Country:			0.001			0.440
Europe	140 (73.3)	302 (84.8)		471 (76.3)	58 (80.6)	
North America	15 (7.8)	21 (5.9)		37 (6.0)	6 (8.3)	
Latin America	4 (2.1)	10 (2.8)		30 (4.9)	3 (4.2)	
Israel	32 (16.8)	23 (6.5)		79 (12.8)	5 (6.9)	
Year at diagnosis:			0.451			0.565
2000-2004	36 (18.8)	84 (23.6)		131 (21.2)	13 (18.1)	
2005-2008	68 (35.6)	118 (33.1)		236 (38.2)	25 (34.7)	
2009-2012	87 (45.5)	154 (43.3)		250 (40.5)	34 (47.2)	
Age at diagnosis, median (IQR) years	35 (31-38)	36 (32-38)	0.244	34 (30-37)	36 (33-38)	0.003
Age at diagnosis:			0.394			<0.001
≤ 30 years	38 (19.9)	58 (16.3)		168 (27.2)	6 (8.3)	
31-35 years	67 (35.1)	118 (33.1)		214 (34.7)	25 (34.7)	
36-40 years	86 (45.0)	180 (50.6)		235 (38.1)	41 (56.9)	
Histology:			0.137			0.107
Ductal carcinoma	150 (78.5)	286 (80.3)		495 (80.2)	60 (83.3)	
Lobular carcinoma	9 (4.7)	28 (7.9)		1 (0.2)	1 (1.4)	
Others	14 (7.4)	15 (4.2)		73 (11.8)	5 (7.0)	
Missing	18 (9.4)	27 (7.6)		48 (7.8)	6 (8.3)	
Tumor grade:			0.043			0.061
G1	6 (3.1)	11 (3.1)		4 (0.6)	2 (2.8)	
G2	50 (26.2)	129 (36.2)		60 (9.7)	11 (15.3)	
G3	125 (65.4)	195 (54.8)		513 (83.1)	56 (77.8)	
Missing	10 (5.2)	21 (5.9)		40 (6.5)	3 (4.2)	
Tumor size:			0.318			0.444
T1 (≤ 2 cm)	89 (46.6)	151 (42.4)		242 (39.2)	25 (34.7)	
T2-T3-T4 (> 2 cm)	98 (51.3)	200 (56.2)		361 (58.5)	47 (65.3)	
Missing	4 (2.1)	5 (1.4)		14 (2.3)	0 (0.0)	
Nodal status:			0.001			0.305
N0	104 (54.4)	143 (40.2)		380 (61.6)	40 (55.6)	
N1-N2-N3	84 (44.0)	209 (58.7)		227 (36.8)	31 (43.0)	
Missing	3 (1.6)	4 (1.1)		10 (1.6)	1 (1.4)	
HER2 status:			0.228			<0.001
HER2 negative	173 (90.6)	319 (89.6)		587 (95.1)	61 (84.7)	
HER2 positive	10 (5.2)	30 (8.4)		18 (2.9)	10 (13.9)	
Missing	8 (4.2)	7 (2.0)		12 (1.9)	1 (1.4)	
Breast surgery:			0.017			0.102
	88 (46.1)	126 (35.4)		340 (55.1)	32 (44.4)	

Breast conserving surgery<br>Mastectomy<br>Missing	101 (52.9)<br>2 (1.0)	225 (63.2)<br>5 (1.4)		271 (43.9)<br>6 (1.0)	39 (54.2)<br>1 (1.4)	
Use of chemotherapy:			0.628			0.363
No	14 (7.3)	31 (8.7)		10 (1.6)	2 (2.8)	
Yes	176 (92.1)	325 (91.3)		606 (98.2)	70 (97.2)	
Missing	1 (0.5)	0 (0.0)		1 (0.2)	0 (0.0)	
Type of chemotherapy*:			0.682			0.987
Anthracycline- and taxane-based	116 (65.9)	215 (66.1)		410 (67.7)	48 (68.6)	
Anthracycline-based	44 (25.0)	91 (28.0)		152 (25.1)	19 (27.1)	
Taxane-based	6 (3.4)	10 (3.1)		14 (2.3)	1 (1.4)	
Others	5 (2.8)	5 (1.5)		14 (2.3)	1 (1.4)	
Missing	5 (2.8)	4 (1.2)		16 (2.6)	1 (1.4)	
Use of endocrine therapy:			<0.001			
No	28 (14.7)	15 (4.2)		NA	NA	NA
Yes	160 (83.8)	339 (95.2)				
Missing	3 (1.6)	2 (0.6)				
Type of endocrine therapy**:			0.379			
Tamoxifen alone	71 (44.4)	145 (42.8)				
Tamoxifen + LHRHa	47 (29.4)	116 (34.2)				
LHRHa alone	5 (3.1)	3 (0.9)		NA	NA	NA
AI ± LHRHa	7 (4.4)	14 (4.1)				
Tamoxifen and AI (± LHRHa)	29 (18.1)	58 (17.1)				
Missing	1 (0.6)	3 (0.9)				
Duration of endocrine therapy, median (IQR) months	60 (36-60)	60 (48-60)	0.049	NA	NA	NA

[a] Calculated after exclusion of missing values

* Calculated among patients who received chemotherapy
** Calculated among patients with hormone receptor-positive breast cancer who received endocrine therapy

Abbreviations: IQR, interquartile range; G, tumor grade; T, tumor size; N, nodal status; ER, estrogen receptor; PR, progesterone receptor; LHRHa, luteinizing hormone-releasing hormone agonists; AI, aromatase inhibitors.

Supplementary Table 3. Use of prophylactic surgery comparing between patients with germline *BRCA1* and *BRCA2* pathogenic variants according to hormone receptor status.

	**Hormone receptor-positive**		**P value[a]**	**Hormone receptor-negative**		**P value[a]**
	***BRCA1* cohort N (%) N=191**	***BRCA2* cohort N (%) N=356**		***BRCA1* cohort N (%) N=617**	***BRCA2* cohort N (%) N=72**	
Prophylactic bilateral risk-reducing mastectomy:			0.774			0.035
No	86 (45.0)	152 (42.7)		268 (43.4)	23 (31.9)	
Yes	82 (42.9)	155 (43.5)		273 (44.2)	42 (58.3)	
Missing	23 (12.0)	49 (13.8)		76 (12.3)	7 (9.7)	
Prophylactic bilateral risk-reducing salpingo-oophorectomy:			0.850			0.796
No	82 (42.9)	147 (41.3)		246 (39.9)	29 (40.3)	
Yes	90 (47.1)	170 (47.7)		300 (48.6)	39 (54.2)	
Missing	19 (9.9)	39 (11.0)		71 (11.5)	4 (5.6)	
Date of prophylactic bilateral risk-reducing salpingo-oophorectomy:						
Not available, N (%)	106 (55.5)	196 (55.1)	0.928	335 (54.3)	36 (50.0)	0.533
Median, months (IQR)*	38.5 (19.5-72.9)	61.2 (27.1-96.3)	0.012	56.8 (21.5-94.2)	63.1 (24.3-98.3)	0.701

[a] Calculated after exclusion of missing values

*among patients with this information available

Supplementary Table 4. Pattern of invasive disease-free survival events comparing between patients with germline *BRCA1* and *BRCA2* pathogenic variants according to hormone receptor status.

	**Hormone receptor-negative**		**P value***	**Hormone receptor-positive**		**P value***
	***BRCA1* cohort N (%) N=617**	***BRCA2* cohort N (%) N=72**		***BRCA1* cohort N (%) N=191**	***BRCA2* cohort N (%) N=356**	
Follow-up, median (IQR)	7.9 (5.6-10.7)	7.7 (5.4-10.3)	0.718	7.6 (5.2-10.4)	8.0 (6.0-10.7)	0.612
No events	377 (61.1)	48 (66.7)	0.323	115 (60.2)	214 (60.1)	0.248
Loco-regional recurrence	40 (6.5)	5 (6.9)	0.835	14 (7.3)	31 (8.7)	0.774
Distant recurrence +/- loco-regional recurrence	56 (9.1)	6 (8.3)	0.804	28 (14.7)	60 (16.9)	0.685
Second primary malignancy	25 (4.1)	3 (4.2)	0.948	10 (5.2)	5 (1.4)	0.005
Ovarian cancer	12 (1.9)	0 (0.0)		6 (3.1)	1 (0.3)	
Other	4 (0.7)	1 (1.4)		3 (1.6)	3 (0.8)	
Missing	9 (1.5)	2 (2.8)		1 (0.5)	1 (0.3)	
Second primary breast cancer	114 (18.5)	9 (12.5)	0.185	23 (12.0)	43 (12.1)	0.579
Death without any disease-free survival event	5 (0.8)	1 (1.4)	0.620	1 (0.5)	3 (0.84)	0.747

*P-values for time-dependent events estimated by means of the Log-rank test

Abbreviations: IQR, interquartile range

Supplementary Table 5. Disease-free survival comparing between patients with germline *BRCA1* and *BRCA2* pathogenic variants according to hormone receptor status.

Disease-Free Survival						
	BRCA1		***BRCA2***			
	Patients/ Events*	Survival 8-years (%) (95% CI)	Patients/ Events*	Survival 8-years (%) (95% CI)	Hazard Ratio* *BRCA2 vs BRCA1* (95% CI)	P-value interaction *BRCA* / HR
**HR-**	617/240	63.7 (59.4 - 67.7)	72/24	73.0 (60.5 - 82.1)	0.73 (0.48 - 1.13)	0.848
**HR+**	191/76	60.0 (51.7 - 67.3)	356/142	64.5 (58.8 - 69.6)	0.77 (0.58 - 1.03)	
**Total**	808/316	62.8 (59.0 - 66.4)	428/166	65.9 (60.8 - 70.5)	0.76 (0.60 - 0.96)	

*: for all period of follow-up; Hazard Ratios adjusted by grade, nodal status, HER2, type of breast surgery, chemotherapy use, age, year of diagnosis and country

Abbreviations: CI, confidence intervals; HR, hazard ratio; HR+, hormone receptor-positive; HR-, hormone receptor-negative.

Supplementary Table 6. Distant recurrence-free interval comparing between patients with germline *BRCA1* and *BRCA2* pathogenic variants according to hormone receptor status.

Distant Recurrence-Free Interval						
	BRCA1		*BRCA2*			
	Patients/ Events*	Survival 8-years (%) (95% CI)	Patients/ Events*	Survival 8-years (%) (95% CI)	Hazard Ratio* *BRCA2 vs BRCA1* (95% CI)	P-value interaction **BRCA** / HR
**HR-**	617/63	90.0 (87.2-92.2)	72/8	91.2 (81.4-95.9)	0.92 (0.43-1.95)	0.946
**HR+**	191/30	85.5 (79.4-89.9)	356/63	82.3 (77.7-86.1)	0.94 (0.60-1.48)	
**Total**	808/93	88.9 (86.4-91.0)	428/71	83.8 (79.7-87.1)	0.94 (0.64-1.38)	

* for all period of follow-up; Hazard Ratios adjusted by grade, nodal status, HER2, type of breast surgery, chemotherapy use, age, year of diagnosis and country

Abbreviations: CI, confidence intervals; HR, hazard ratio; HR+, hormone receptor-positive; HR-, hormone receptor-negative.

Supplementary Table 7. Overall survival comparing comparing between patients with germline *BRCA1* and *BRCA2* pathogenic variants according to hormone receptor status.

Overall Survival						
	BRCA1		***BRCA2***			
	Patients/ Events*	Survival 8-years (%) (95% CI)	Patients/ Events*	Survival 8-years (%) (95% CI)	Hazard Ratio* *BRCA2 vs BRCA1* (95% CI)	P-value interaction *BRCA* / HR
**HR-**	617/86	86.9 (83.8 - 89.5)	72/10	87.2 (75.9 - 93.5)	0.80 (0.40 - 1.56)	0.624
**HR+**	191/27	86.9 (80.6 - 91.2)	356/46	87.6 (83.1 - 91.0)	0.64 (0.39 - 1.05)	
**Total**	808/113	86.9 (84.2 - 89.2)	428/56	87.5 (83.5 - 90.7)	0.69 (0.46 - 1.04)	

*: for all period of follow-up; Hazard Ratios adjusted by grade, nodal status, HER2, type of breast surgery, chemotherapy use, age, year of diagnosis and country

Abbreviations: CI, confidence intervals; HR, hazard ratio; HR+, hormone receptor-positive; HR-, hormone receptor-negative.

Supplementary Table 8. Patient, tumor and treatment characteristics comparing between patients with hormone receptor-positive and negative breast cancer.

	Hormone receptor-positive N (%) N=547	Hormone receptor-negative N (%) N=689	P value[a]
Country:			0.115
Europe	442 (80.8)	529 (76.8)	
North America	36 (6.6)	43 (6.2)	
Latin America	14 (2.6)	33 (4.8)	
Israel	55 (10.0)	84 (12.2)	
Year at diagnosis:			0.367
2000-2004	120 (21.9)	144 (20.9)	
2005-2008	186 (34.0)	261 (37.9)	
2009-2012	241 (44.1)	284 (41.2)	
Age at diagnosis, median (IQR) years	35 (32-38)	34 (30-37)	0.003
Age at diagnosis:			0.001
≤ 30 years	96 (17.5)	174 (25.2)	
31-35 years	185 (33.8)	239 (34.7)	
36-40 years	266 (48.6)	276 (40.1)	
Histology:			<0.001
Ductal carcinoma	436 (79.7)	555 (80.5)	
Lobular carcinoma	37 (6.8)	2 (0.3)	
Others	29 (5.3)	78 (11.3)	
Missing	45 (8.2)	54 (7.8)	
Tumor grade:			<0.001
G1	17 (3.1)	6 (0.9)	
G2	179 (32.7)	71 (10.3)	
G3	320 (58.5)	569 (82.6)	
Missing	31 (5.7)	43 (6.2)	
Tumor size:			0.079
T1 (≤ 2 cm)	240 (43.9)	267 (38.7)	
T2-T3-T4 (> 2 cm)	298 (54.5)	408 (59.2)	
Missing	9 (1.6)	14 (2.0)	
Nodal status:			<0.001
N0	247 (45.2)	420 (61.0)	
N1-N2-N3	293 (53.6)	258 (37.4)	
Missing	7 (1.3)	11 (1.6)	
BRCA cohort:			<0.001
BRCA1	191 (34.9)	617 (89.5)	
BRCA2	356 (65.1)	72 (10.5)	
HER2 status:			0.016
HER2 negative	492 (90.0)	648 (94.0)	
HER2 positive	40 (7.3)	28 (4.1)	
Missing	15 (2.7)	13 (1.9)	
Breast surgery:			<0.001
Breast conserving surgery	214 (39.1)	372 (54.0)	

Mastectomy<br>Missing	326 (59.6)<br>7 (1.3)	310 (45.0)<br>7 (1.0)	
Use of chemotherapy:<br>No<br>Yes<br>Missing	 45 (8.2)<br>501 (91.6)<br>1 (0.2)	 12 (1.7)<br>676 (98.1)<br>1 (0.2)	<0.001
Type of chemotherapy*:<br>Anthracycline- and taxane-based<br>Anthracycline-based<br>Taxane-based<br>Others<br>Missing	 331 (60.5)<br>135 (24.7)<br>16 (2.9)<br>10 (1.8)<br>51 (9.3)	 458 (66.5)<br>171 (24.8)<br>15 (2.2)<br>15 (2.2)<br>26 (3.8)	0.676
Use of endocrine therapy**:<br>No<br>Yes<br>Missing	 43 (7.9)<br>499 (91.2)<br>5 (0.9)	NA	NA
Type of endocrine therapy***:<br>Tamoxifen alone<br>Tamoxifen + LHRHa<br>LHRHa alone<br>AI ± LHRHa<br>Tamoxifen and AI (± LHRHa)<br>Missing	 216 (43.3)<br>163 (32.7)<br>8 (1.6)<br>21 (4.2)<br>87 (17.4)<br>4 (0.8)	NA	NA
Duration of endocrine therapy, median (IQR) months	60 (43.5-60)	NA	NA

[a] Calculated after exclusion of missing values

* Calculated among patients who received chemotherapy
** Calculated among patients with hormone receptor-positive breast cancer
*** Calculated among patients with hormone receptor-positive breast cancer who received endocrine therapy

Abbreviations: IQR, interquartile range; G, tumor grade; T, tumor size; N, nodal status; ER, estrogen receptor; PR, progesterone receptor; LHRHa, luteinizing hormone-releasing hormone agonists; AI, aromatase inhibitors.

Supplementary Table 9. Use of prophylactic surgery comparing between patients with hormone receptor-positive and negative breast cancer.

	**Hormone receptor-positive N (%) N=547**	**Hormone receptor-negative N (%) N=689**	**P value**
Prophylactic bilateral risk-reducing			0.501
mastectomy:	238 (43.5)	291 (42.2)	
No	237 (43.3)	315 (45.7)	
Yes	72 (13.2)	83 (12.1)	
Missing			
Prophylactic bilateral risk-reducing			0.504
salpingo-oophorectomy:			
No	229 (41.9)	275 (39.9)	
Yes	260 (47.5)	339 (49.2)	
Missing	58 (10.6)	75 (10.9)	
Date of prophylactic bilateral risk-reducing salpingo-oophorectomy:			
Not available, N (%)	302 (55.2)	371 (53.9)	0.646
Median, months (IQR)*	50.3 (22.6-88.8)	56.8 (21.8-94.2)	0.879

[a] Calculated after exclusion of missing values

*among patients with this information available

Supplementary Table 10. Survival outcomes comparing between patients with hormone receptor-positive and negative breast cancer.

	**Hormone receptor-positive**		**Hormone receptor-negative**		
	Patients/ Events*	Survival 8-years (%) (95% CI)	Patients/ Events*	Survival 8-years (%) (95% CI)	Hazard Ratio* HR+ vs HR- (95% CI)
**DFS**	547/218	62.9 (58.3-67.2)	689/264	64.7 (60.6-68.4)	1.06 (0.84-1.33)
**DRFI**	547/93	83.4 (79.8-86.4)	689/71	90.1 (87.5-92.2)	1.39 (0.94-2.05)
**OS**	547/73	87.3 (83.7-90.1)	689/96	87.0 (84.0-89.4)	0.81 (0.55-1.20)

*: for all period of follow-up; Hazard Ratios adjusted by grade, nodal status, HER2, type of breast surgery, chemotherapy use, age, year of diagnosis and country

Abbreviations: DFS, disease-free survival; DRFI, distant recurrence-free interval; OS, overall survival; CI, confidence intervals; HR+, hormone receptor-positive; HR-, hormone receptor-negative.

Supplementary Table 11. Patient, tumor and treatment characteristics comparing between patients with hormone receptor-positive and negative breast cancer (by including only patients for whom the 1% cut-off for estrogen and/or progesterone receptor expression in their tumor was used to define hormone receptor status).

	**Hormone receptor-positive N (%) N=385**	**Hormone receptor-negative N (%) N=493**	**P value[a]**
Country:			0.124
Europe	280 (72.7)	333 (67.6)	
North America	36 (9.4)	43 (8.7)	
Latin America	14 (3.6)	33 (6.7)	
Israel	55 (14.3)	84 (17.0)	
Year at diagnosis:			0.273
2000-2004	91 (23.6)	115 (23.3)	
2005-2008	124 (32.2)	183 (37.1)	
2009-2012	170 (44.2)	195 (39.6)	
Age at diagnosis, median (IQR) years	35 (32-38)	34 (31-37)	0.044
Age at diagnosis:			0.016
≤ 30 years	66 (17.1)	122 (24.7)	
31-35 years	135 (35.1)	170 (34.5)	
36-40 years	184 (47.8)	201 (40.8)	
Histology:			<0.001
Ductal carcinoma	287 (74.6)	378 (76.7)	
Lobular carcinoma	26 (6.7)	2 (0.4)	
Others	27 (7.0)	61 (12.4)	
Missing	45 (11.7)	52 (10.5)	
Tumor grade:			<0.001
G1	12 (3.1)	5 (1.0)	
G2	128 (33.2)	48 (9.7)	
G3	217 (56.4)	404 (82.0)	
Missing	28 (7.3)	36 (7.3)	
Tumor size:			0.071
T1 (≤ 2 cm)	173 (44.9)	189 (38.4)	
T2-T3-T4 (> 2 cm)	207 (53.8)	293 (59.4)	
Missing	5 (1.3)	11 (2.2)	
Nodal status:			<0.001
N0	161 (41.8)	292 (59.2)	
N1-N2-N3	217 (56.4)	191 (38.8)	
Missing	7 (1.8)	10 (2.0)	
BRCA cohort:			<0.001
BRCA1	142 (36.9)	434 (88.0)	
BRCA2	243 (63.1)	59 (12.0)	
HER2 status:			0.005
HER2 negative	339 (88.1)	462 (93.7)	
HER2 positive	31 (8.0)	18 (3.7)	
Missing	15 (3.9)	13 (2.6)	

Breast surgery:			0.001
Breast conserving surgery	147 (38.2)	245 (49.7)	
Mastectomy	231 (60.0)	243 (49.3)	
Missing	7 (1.8)	5 (1.0)	
Use of chemotherapy:			<0.001
No	40 (10.4)	11 (2.2)	
Yes	344 (89.3)	481 (97.6)	
Missing	1 (0.3)	1 (0.2)	
Type of chemotherapy*:			0.748
Anthracycline- and taxane-based	225 (65.4)	311 (64.7)	
Anthracycline-based	88 (25.6)	129 (26.8)	
Taxane-based	13 (3.8)	12 (2.5)	
Others	9 (2.6)	13 (2.7)	
Missing	9 (2.6)	16 (3.3)	
Use of endocrine therapy**:			
No	29 (7.5)	NA	NA
Yes	351 (91.2)		
Missing	5 (1.3)		
Type of endocrine therapy***:			
Tamoxifen alone	122 (34.7)		
Tamoxifen + LHRHa	144 (41.0)		
LHRHa alone	7 (2.0)	NA	NA
AI ± LHRHa	14 (4.0)		
Tamoxifen and AI (± LHRHa)	62 (17.7)		
Missing	2 (0.6)		
Duration of endocrine therapy, median (IQR) months	60 (47-60)	NA	NA

[a] Calculated after exclusion of missing values

* Calculated among patients who received chemotherapy
** Calculated among patients with hormone receptor-positive breast cancer
*** Calculated among patients with hormone receptor-positive breast cancer who received endocrine therapy

Abbreviations: IQR, interquartile range; G, tumor grade; T, tumor size; N, nodal status; ER, estrogen receptor; PR, progesterone receptor; LHRHa, luteinizing hormone-releasing hormone agonists; AI, aromatase inhibitors.

Supplementary Table 12. Use of prophylactic surgery comparing between patients with hormone receptor-positive and negative breast cancer (by including only patients for whom the 1% cut-off for estrogen and/or progesterone receptor expression in their tumor was used to define hormone receptor status).

	**Hormone receptor-positive N (%) N=385**	**Hormone receptor-negative N (%) N=493**	**P value**
Prophylactic bilateral risk-reducing mastectomy:			0.530
No	183 (47.5)	229 (46.5)	
Yes	179 (46.5)	246 (49.9)	
Missing	23 (6.0)	18 (3.6)	
Prophylactic bilateral risk-reducing salpingo-oophorectomy:			0.678
No	167 (43.4)	221 (44.8)	
Yes	208 (54.0)	259 (52.6)	
Missing	10 (2.6)	13 (2.6)	
Date of prophylactic bilateral risk-reducing salpingo-oophorectomy:			
Not available, N (%)	191(49.6)	256 (51.9)	0.497
Median, months (IQR)*	51.2 (20.5-92.2)	59.5 (19.5-99.2)	0.504

[a] Calculated after exclusion of missing values

*among patients with this information available

Table 13. Pattern of invasive disease-free survival events according to hormone receptor status (by including only patients for whom the 1% cut-off for estrogen and/or progesterone receptor expression in their tumor was used to define hormone receptor status).

	**Hormone receptor-positive N (%) n=385 (56.1)**	**Hormone receptor-negative N (%) n=493 (43.9)**	***P* value[a]**
Follow-up, median (IQR)	8.0 (6.0-10.9)	8.0 (5.6-10.9)	0.600
No events	230 (59.7)	309 (62.7)	0.566
Loco-regional recurrence	25 (6.5)	27 (5.5)	0.562
Distant recurrence +/- loco-regional recurrence	69 (17.9)	41 (8.3)	<0.001
Second primary malignancy	11 (2.9)	19 (3.9)	0.361
Ovarian cancer	6 (1.6)	7 (1.4)	
Other	3 (0.8)	5 (1.1)	
Missing	2 (0.5)	7 (1.4)	
Second primary breast cancer	48 (12.5)	91 (18.5)	0.014
Death without any disease-free survival event	2 (0.5)	6 (1.2)	0.257

[a] P-values for time-dependent events estimated by means of the Log-rank test

Abbreviations: IQR, interquartile range.

Supplementary Table 14. Survival outcomes comparing between patients with hormone receptor-positive and negative breast cancer (by including only patients for whom the 1% cut-off for estrogen and/or progesterone receptor expression in their tumor was used to define hormone receptor status).

	**Hormone receptor-positive**		**Hormone receptor-negative**		
	Patients/ Events*	Survival 8-years (%) (95% CI)	Patients/ Events*	Survival 8-years (%) (95% CI)	Hazard Ratio* HR+ vs HR- (95% CI)
**DFS**	385/155	63.7 (58.2-68.6)	493/184	66.8 (62.0-71.0)	1.01 (0.77-1.33)
**DRFI**	385/71	82.3 (77.8-85.9)	493/49	90.7 (87.6-93.1)	1.53 (0.98-2.40)
**OS**	385/56	85.8 (81.5-89.2)	493/70	86.8 (83.2-89.6)	0.88 (0.56-1.37)

*: for all period of follow-up; Hazard Ratios adjusted by grade, nodal status, HER2, type of breast surgery, chemotherapy use, age, year of diagnosis and country

Abbreviations: DFS, disease-free survival; DRFI, distant recurrence-free interval; OS, overall survival; CI, confidence intervals; HR+, hormone receptor-positive; HR-, hormone receptor-negative.

Supplementary Figure 2. Comparison between patients with hormone receptor-positive and negative disease: Epanechnikov Kernel-Smoothed annual hazards of recurrence overall.

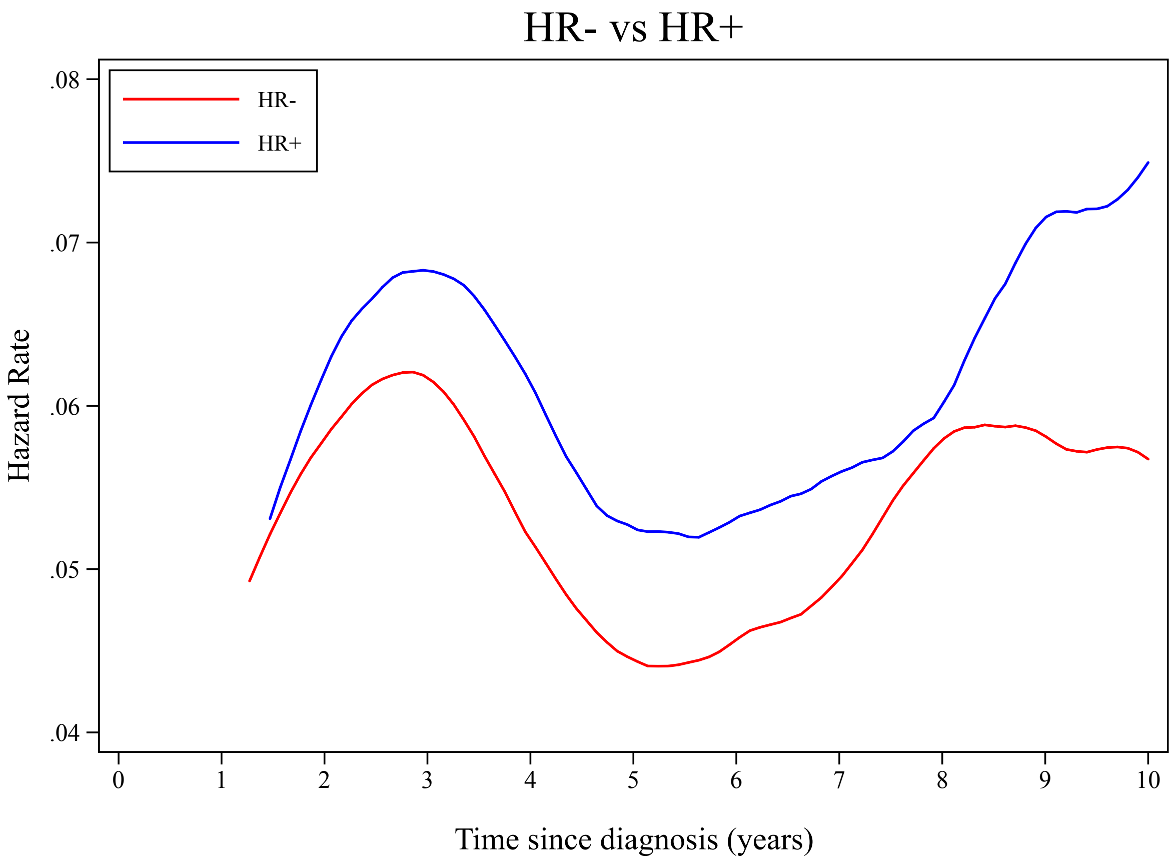

HR+ = hormone receptor-positive; HR- = hormone receptor-negative.

Supplementary Figure 3. Comparison between patients with hormone receptor-positive and negative disease: Disease-free survival.

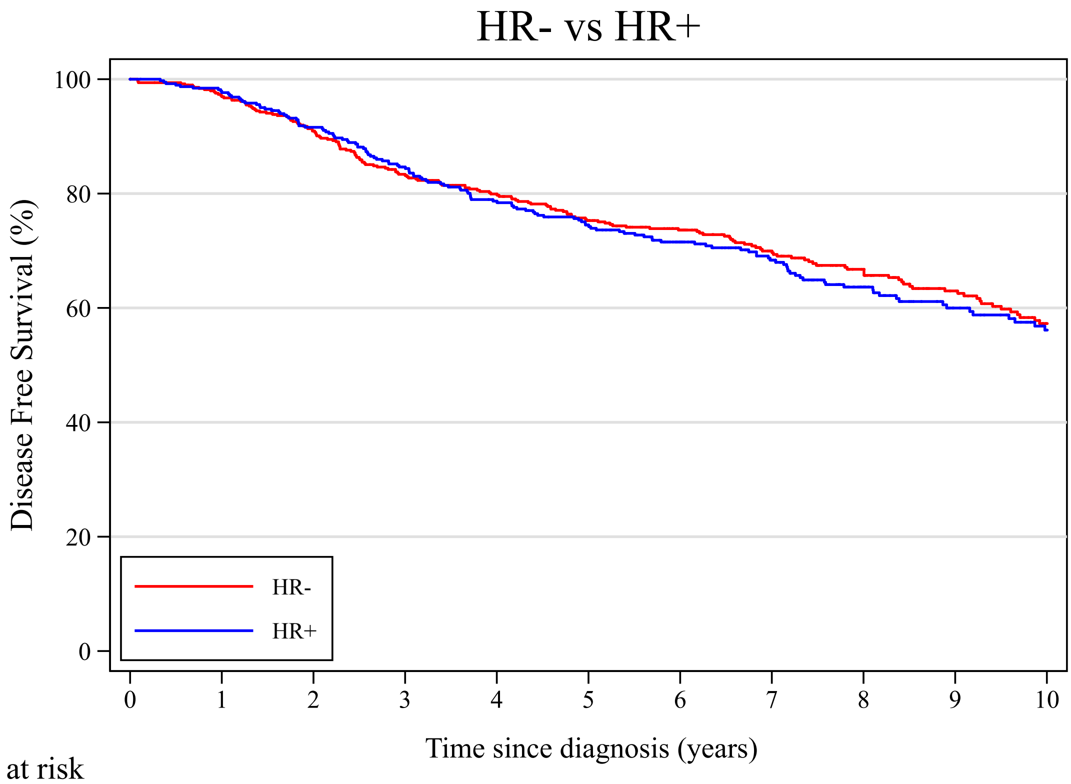

Number at risk

HR-	493	473	435	393	368	333	288	231	190	150	106
HR+	385	375	344	315	286	261	221	186	135	101	80

HR+ = hormone receptor-positive; HR- = hormone receptor-negative.

Supplementary Figure 4. Comparison between patients with hormone receptor-positive and negative disease: Distant recurrence-free interval.

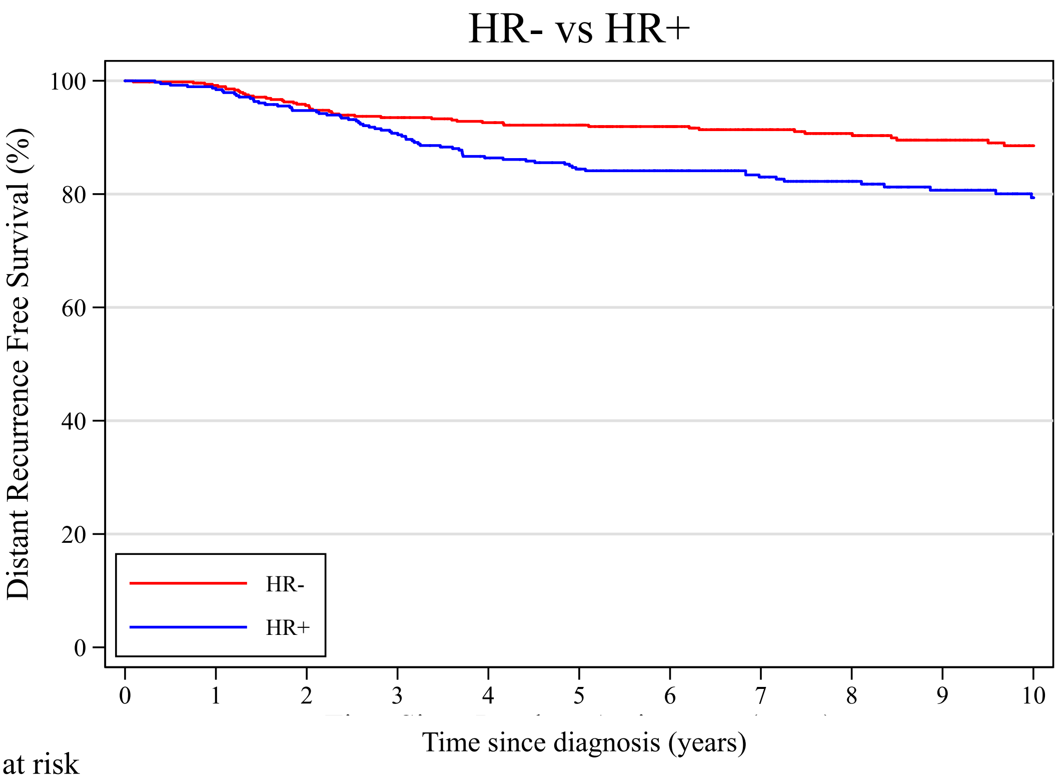

Number at risk

HR-	493	480	450	430	413	394	344	293	247	204	163
HR+	385	378	355	337	312	294	259	224	176	139	112

HR+ = hormone receptor-positive; HR- = hormone receptor-negative.

Supplementary Figure 5. Comparison between patients with hormone receptor-positive and negative disease: Overall survival.

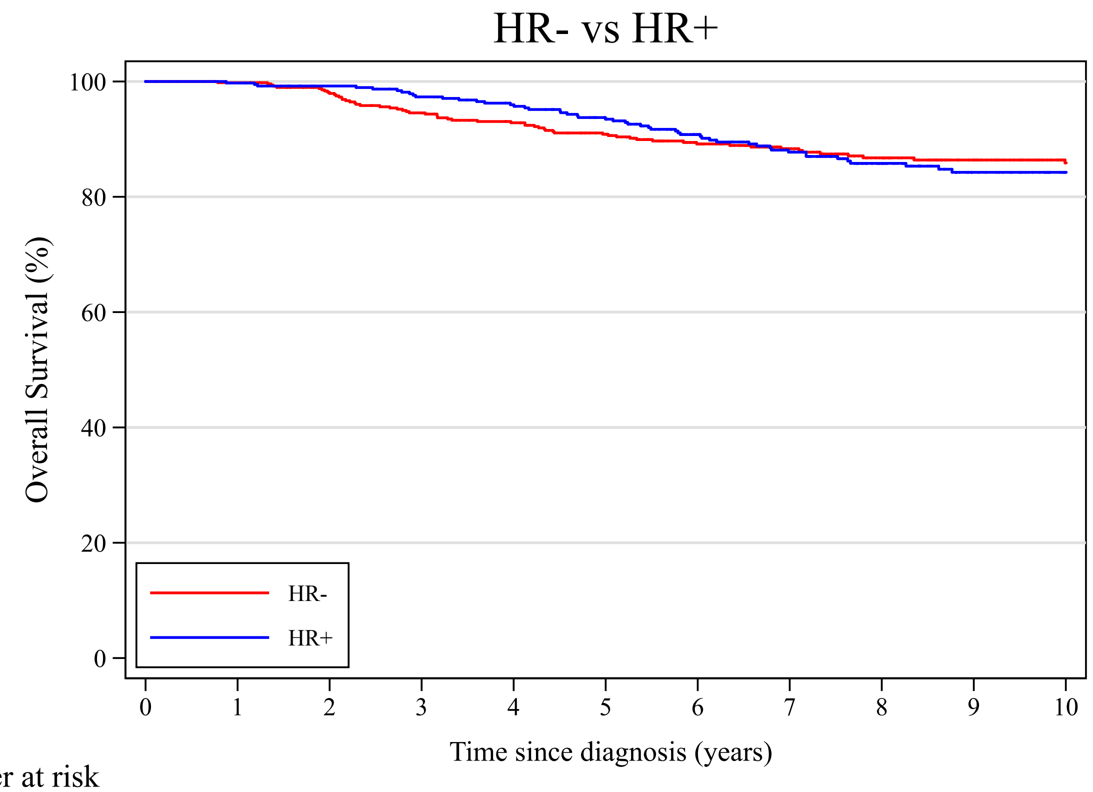

Number at risk											
HR-	493	486	467	445	426	401	350	298	251	209	169
HR+	385	382	371	361	348	329	286	244	192	149	124

HR+ = hormone receptor-positive; HR- = hormone receptor-negative.
