# Supplementary material for: Clinical behavior and outcomes of breast cancer in young women with germline BRCA pathogenic variants
Source: NPJ Breast Cancer. 2021 Feb 12;7:16. doi: 10.1038/s41523-021-00224-w (PMC7880991; doi:10.1038/s41523-021-00224-w)
Supplement: Supplementary file 2 — Reporting Summary Checklist [file 41523_2021_224_MOESM2_ESM.pdf]

nature research

Corresponding author(s): Matteo Lambertini

Last updated by author(s): Dec 2, 2020

Reporting Summary

Nature Research wishes to improve the reproducibility of the work that we publish. This form provides structure for consistency and transparency in reporting. For further information on Nature Research policies, see our Editorial Policies and the Editorial Policy Checklist.

Statistics

For all statistical analyses, confirm that the following items are present in the figure legend, table legend, main text, or Methods section.

n/a	Confirmed	
☐	☒	The exact sample size (*n*) for each experimental group/condition, given as a discrete number and unit of measurement
☐	☒	A statement on whether measurements were taken from distinct samples or whether the same sample was measured repeatedly
☐	☒	The statistical test(s) used AND whether they are one- or two-sided<br>*Only common tests should be described solely by name; describe more complex techniques in the Methods section.*
☐	☒	A description of all covariates tested
☐	☒	A description of any assumptions or corrections, such as tests of normality and adjustment for multiple comparisons
☐	☒	A full description of the statistical parameters including central tendency (e.g. means) or other basic estimates (e.g. regression coefficient) AND variation (e.g. standard deviation) or associated estimates of uncertainty (e.g. confidence intervals)
☒	☐	For null hypothesis testing, the test statistic (e.g. *F*, *t*, *r*) with confidence intervals, effect sizes, degrees of freedom and *P* value noted<br>*Give P values as exact values whenever suitable.*
☒	☐	For Bayesian analysis, information on the choice of priors and Markov chain Monte Carlo settings
☒	☐	For hierarchical and complex designs, identification of the appropriate level for tests and full reporting of outcomes
☒	☐	Estimates of effect sizes (e.g. Cohen's *d*, Pearson's *r*), indicating how they were calculated

Our web collection on statistics for biologists contains articles on many of the points above.

Software and code

Policy information about availability of computer code

Data collection	Reported in the study methods
Data analysis	Statistical analyses were performed by MC and MB using Stata 13.1 (StataCorp. 2013. Stata Statistical Software: Release 13. College Station, TX: StataCorp LP).

For manuscripts utilizing custom algorithms or software that are central to the research but not yet described in published literature, software must be made available to editors and reviewers. We strongly encourage code deposition in a community repository (e.g. GitHub). See the Nature Research guidelines for submitting code & software for further information.

Data

Policy information about availability of data

All manuscripts must include a data availability statement. This statement should provide the following information, where applicable:

- Accession codes, unique identifiers, or web links for publicly available datasets
- A list of figures that have associated raw data
- A description of any restrictions on data availability

The dataset analyzed during this study is described with more details in the following manuscript: doi 10.1200/JCO.19.02399 39. The data used for the present analysis are not publicly available, but they can be made available to researchers upon reasonable request addressed to the corresponding author of the present manuscript.

Field-specific reporting

Please select the one below that is the best fit for your research. If you are not sure, read the appropriate sections before making your selection.

☒ Life sciences ☐ Behavioural & social sciences ☐ Ecological, evolutionary & environmental sciences

For a reference copy of the document with all sections, see nature.com/documents/nr-reporting-summary-flat.pdf

Life sciences study design

All studies must disclose on these points even when the disclosure is negative.

Sample size	A total of 1,236 young BRCA-mutated breast cancer patients were eligible for inclusion in the present analysis
Data exclusions	Considering that the cut-off used for defining hormone receptor positivity was not homogenous in all centers, the analyses comparing between patients with hormone receptor-positive and negative disease were then repeated by including only patients for whom the 1% cut-off for estrogen and/or progesterone receptor expression in their tumor was used to define hormone receptor status. Results were consistent to those reported in the main analyses (Supplementary Tables 11-14 and Supplementary Figures 2-5, available online).
Replication	Not applicable
Randomization	Not applicable
Blinding	Not applicable

Reporting for specific materials, systems and methods

We require information from authors about some types of materials, experimental systems and methods used in many studies. Here, indicate whether each material, system or method listed is relevant to your study. If you are not sure if a list item applies to your research, read the appropriate section before selecting a response.

Materials & experimental systems

n/a	Involved in the study	
☒	☐	Antibodies
☒	☐	Eukaryotic cell lines
☒	☐	Palaeontology and archaeology
☒	☐	Animals and other organisms
☐	☒	Human research participants
☐	☒	Clinical data
☒	☐	Dual use research of concern

Methods

n/a	Involved in the study	
☒	☐	ChIP-seq
☒	☐	Flow cytometry
☒	☐	MRI-based neuroimaging

Human research participants

Policy information about studies involving human research participants

Population characteristics	This was an international, multicenter, hospital-based, retrospective cohort study that included women diagnosed at age ≤ 40 years with invasive early breast cancer (stage I-III) between January 2000 and December 2012. All included patients had a known germline BRCA1 or BRCA2 pathogenic variant.<br>Healthy carriers as well as women with BRCA variants of uncertain significance, other malignancies (including ovarian cancer) without prior diagnosis of invasive breast cancer, in situ or stage IV de novo breast cancer, or lack of information on follow-up were not eligible for inclusion. For the purpose of the present analysis, patients harboring pathogenic variants in both BRCA1 and BRCA2 as well as those with unknown hormone receptor status were also excluded.
Recruitment	This was an international, multicenter, hospital-based, retrospective cohort study that included women diagnosed at age ≤ 40 years with invasive early breast cancer (stage I-III) between January 2000 and December 2012. All included patients had a known germline BRCA1 or BRCA2 pathogenic variant.
Ethics oversight	The Institut Jules Bordet (Brussels, Belgium) coordinated the study and acted as central ethics committee. Ethics approval by the Institutional Review Boards of participating centers and patients' written informed consent were obtained before inclusion whenever requested by local regulations.

Note that full information on the approval of the study protocol must also be provided in the manuscript.

Clinical data

Policy information about clinical studies

All manuscripts should comply with the ICMJE guidelines for publication of clinical research and a completed CONSORT checklist must be included with all submissions.

Clinical trial registration	ClinicalTrials.gov Identifier: NCT03673306
Study protocol	Not applicable
Data collection	Data on tumor and patient characteristics, treatment, BRCA pathogenic variants and survival outcomes were collected for all eligible patients.<br>The type of mutated gene was the criteria used to distinguish between two cohorts of patients: women with BRCA1 (BRCA1 cohort) and those with BRCA2 (BRCA2 cohort) pathogenic variants.<br>BRCA pathogenic variants and hormone receptor status were assessed locally at each participating center. Hormone receptor positivity was defined by the expression of estrogen and/or progesterone receptors in ≥1% of invasive tumor cells (≥10% in French participating centers) assessed by immunostaining.
Outcomes	The current analysis aimed to investigate the impact of type of mutated gene (BRCA1 vs. BRCA2) and hormone receptor status on clinical behavior and outcomes of young breast cancer patients with germline BRCA pathogenic variants.<br>Clinicopathological characteristics, pattern and risk over time of disease-free survival (DFS) events, as well as prognosis (in terms of DFS, distant recurrence-free interval [DRFI] and overall survival [OS]) were compared between the BRCA1 and BRCA2 cohorts. The same analyses comparing the BRCA1 and BRCA2 cohorts were then performed separately in patients with hormone receptor-positive and negative disease.<br>To specifically assess the prognostic effect of hormone receptor status, clinicopathological characteristics, pattern and risk over time of DFS events, as well as prognosis (in terms of DFS, DRFI and OS) were compared between patients with hormone receptor-positive and negative disease irrespective of type of mutated gene.
